# Extract from the Minutes of the Atlanta Academy of Medicine

**Published:** 1871-10

**Authors:** J. T. Johnson

**Affiliations:** Reporter


### EXTRACT FROM THE MINUTES OF THE ATLANTA ACADEMY OF MEDICINE.

J. T. JOHNSON, M.D., REPORTER.

AUGUST 18th, 1871.

The President, Dr. J. P. Logan, in the chair.

Dr. Logan called attention to his recent successful use of Meigs' ring pessary in a case of prolapsus uteri, in which there had been most extraordinary constitutional disturbance, such as excessive palpitation of the heart, and a train of hysterical symptoms of a most obstinate character, apparently attributable to the displacement. His experience with pessaries generally had been a very unsatisfactory one—so much so as to almost induce him to abandon their use entirely. He had been led to use the ring recently upon the report of Dr. Wm. Henry Cumming, of a most satisfactory and successful experience with that instrument. It seemed, in this instance, to have relieved the unpleasant constitutional symptoms more decidedly than any other means used in the case.

Dr. Cumming stated that he was fully persuaded that sufficient importance was not assigned to the maintenance of the womb at its proper height. A descent of one inch often produces distressing sensations, and in married women, exposes the womb to injurious pressure and percussion during the sexual act — this percussion resulting in tenderness, pain, swelling and ulceration.

The relief often afforded in such cases by merely raising the womb on the tips of the fingers is very great. The woman should be first examined while standing, and the womb be elevated. The woman then lying down, a ring pessary of as large a size as possible should then be introduced. It is a good plan to compress the ring into an elliptical shape by tying firmly with tape, a slip-knot being used. After introducing the pessary, the knot may be readily loosed by pulling upon the free end of the tape. The ring will then resume its circular form, and may be fully adjusted. The woman should then stand up and "bear down" so as to press the womb downward, the physician aiding her efforts by firm pressure upon the hypogastric region. If the womb retains a sufficient elevation notwithstanding these efforts, there is good reason to expect a successful result. If the womb should descend, a larger pessary should be used. Vaginal injections of soap and water should be used once or twice a day while the pessary remains. In women of vigorous structure, ten days will be required, in feeble women, two or three weeks, before the pessary may be safely removed. If there be constipation, enemata must be used to empty the rectum. The bladder, too, must not be allowed to become distended. There need be no interference with the sexual act, nor with the functions of menstruation. Usually, the patient does not feel the pessary, and, if her womb be properly supported, finds herself freed from the distressing sensations previously existing. Many ulcerations of the neck of the womb get well under this treatment, without any local applications whatsoever.

Dr. W. F. Westmoreland does not believe that there is any definite fixed position of the uterus after marriage. Thinks that the descent of the womb has not much to do in causing the train of nervous symptoms that we often find accompanying it, and that are generally believed to be the result of the displacement. Has frequently seen cases in which prolapse had advanced even to procidentia, and yet there was none of these nervous symptons in question. On the other hand, has seen cases where the womb was high up, almost out of reach of the finger, and accompanied with congestion, in which there were present all of those symptoms held by some to be attributable to displacement. Hence, to say the least, the symptoms do not always depend on the descent. Recollects a lecture, years ago, in which the physician took the ground that there was no normal position of the womb, and that these symptoms do not depend on the displacement. Still, has no doubt that frequent prolapse will produce the train of symptoms. Was educated at the Jefferson Medical College, and received the teachings of Meigs, who is a greater pessary-man than even is Dr. Cumming. So impressed was he with his views, so impressed with their correctness, that he wrote his thesis on "Prolapsus Uteri." Now thinks that Meigs was wrong in many, if not in all, of these cases. Has not used a pessary in ten years, though he may be extreme in his opinion, for some are doubtless benefited by support. Cites the authority, which he believes to be very high, of Dr. Warren Stone, who does not use the pessary to correct displacement. If you withdraw the pessary, the support is gone. He (Stone) treats these cases by placing the patient on her back for a number of days, and directing attention to constringing the vagina, and thus give a support to the uterus by the increased tone of this tube. This he effects by a piece of cotton saturated with tannin, or alum, or oak-bark, or other like remedy; uses no pessary, but merely keeps the patient on her back.

Dr. W. H. Cumming.—In regard to the physiological position of the womb, thinks that it has a definite and fixed one. A descent of one inch is a change from its natural position, and will cause a set of nervous phenomena. Some women, though, are as insensible as men—are not more nervous than are men. It is important that the womb must have a definite position; this is essential during married life. So fixed is the organ, that there is not, in a year, as much movement as one-half or even one-fourth of an inch, unless impregnated. We frequently find, in practice, cases in which the womb may be reached by passing the finger into the vagina not to half of its length; we find in them this train of symptoms. Put it up with the pessary, and there is an end of the misery; the patient is able to take exercise, is able to walk the next day. And in this way is the general health improved. You may do nothing else, and in a short while, in a few days, the improvement is apparent to every one. In this way, the elongated ligaments are allowed to retract and regain their natural tone and position. The patient should be always examined in the standing position; though it may be necessary to examine her while recumbent, yet we should not fail to examine in the erect also. If the patient should meet any mishap, as a congestion of the womb, it may descend again, impelled by the increased weight. If the womb comes down, we frequently find pain just behind the pubes, indicating the stretching of the round ligaments. This pain ceases when the tension is relieved by the replacement of the womb.

Dr. Wilson thinks we may be mistaken in attributing this train of symptoms to the displacement, but there has not been proof adduced to-night sufficient to substantiate these views. Slight descent gives more trouble, more nervous symptoms, than procidentia. Has seen the latter, indeed, when the nervous system seemed to take no cognizance whatever of the derangement. But still, this is no evidence, for it may be accounted for by the removal of the pressure from the neighboring tissues when the womb has passed below the pelvis. *Descensus* is more troublesome than procidentia. Has a friend in Texas, a physician and a minister, an eminent man, one of character, who thought it was important to frequently replace the womb. He would visit the patient two or three times in the day for that purpose, so avoiding the use of the pessary.

Dr. H. V. M. Miller would be glad if he were able to remove the doubt from the subject. There is much concerning it about which we are ignorant; but there are some things which he does know. Knows that there is often but slight disturbance from displacement of the womb. Has seen every degree of prolapse in which this was the case, from the slightest malposition to complete procidentia. Take, for illustration, one thousand women: many of them, doubtless, suffer from a variation from what might be called the normal position—or at least suffer from a change of its position—and yet there is no symptom to indicate it; so we are not aware of the malposition. 'Tis only by the presence of these symptoms that we are made aware of its existence; if they are wanting, there arises no necessity for the examination by which alone the true condition of the organ is discovered. Must differ from Dr. Cumming in regard to the support of the ligaments. None of the ligaments are capable of supporting the womb after they are once stretched by pregnancy. They may prevent retroversion; the broad ligament cannot do even that. The support of the womb is not obtained in that way. It is prevented from dropping down by the resistance of the vagina. If that tube or pillar is not weakened, dilated or relaxed, it is of itself adequate to the proper retention. But to say that one-fourth of an inch constitutes a change from which nervous symptoms may ensue, is too close a calculation; we cannot appreciate so slight a change. Recognizes a "normal," but not a "fixed" position. But still we are in doubt as to the true nature of these troubles, and the extent to which the symptoms depend on the displacement; hence, must treat the condition, not forgetting the doubt. Support the vagina and perineum externally. He never uses a band to go around the body for the attachment of this support, but passes the belt so that it may take its point of departure from the shoulder. Has seen this of itself, with but even a wash or other adjuvant, as completely successful as the ring pessary in the hands of Dr. Cumming.

It is a common opinion, among women, that "falling of the womb" is a serious affair. The physician examines, finds displacement, falls into error as did the patient, and attributes, perhaps wrongly, the whole trouble to that displacement. If such is the case, then the ring pessary should cure in every instance. But we all know that Dr. Meigs didn't succeed so well as this with his pessaries. Then, we must treat the disease that gave rise to the prolapse, or build the constitution, and thus give tone to the tissues implicated as best we may.

The train of symptoms under discussion depend on a variety of causes, and not on prolapse alone. Has seen them all present, and yet there was no prolapse. Even if we find some descent, we cannot be always sure that we have found the source of trouble. Finally, we cannot depend on the ring pessary alone, or any other one remedy, for the cure.

---

SEPTEMBER 8th, 1861.

The President, Dr. Logan, in the chair.

The Committee for considering the question as to the antagonism between opium and belladonna, through their Chairman, Dr. Judson, stated that they had been conducting some experiments, but were not yet prepared for a report.

Further time was granted.

Dr. J. W. Vance reports a case of ovarian tumor, in which he had tapped the largest cyst, and drew off more than a quart of fluid, followed by relief, for two or three days, from the attending dyspnœa. The patient was too weak for ovariotomy. The legs were œdematous; had to be propped up to sleep; pressure on diaphragm caused the dyspnœa. Tapped under the mistaken diagnosis of ascites. Filled again very rapidly, and thinks the patient died quicker than she would have otherwise done. Attempted to tap a second time, but from some cause—probably entering another and smaller cyst—only drew out an ounce of fluid, and that of a coagulable nature. Desires the experience of some of the members as to ovariotomy, or tapping.

Dr. Wells.—Was only one side distended?

Dr. Vance.—One principally so.

Dr. Wells.—Does not think that the tapping hastened the final result. The breathing was already accelerated; the capillary circulation was interfered with; the blood probably but poorly oxygenated, and great exhaustion was present.

Dr. Vance.—Would mention that there was present the systolic murmur of the heart that so often attends anæmia.

Dr. Baird.—The dyspnœa might arise from a disease of the heart, rather than from the tumor.

Dr. Vance.—Is sure that the dyspnœa arose from the tumor, and the murmur from anæmia, rather than disease of the heart. Has frequently observed, in hospitals, this murmur in diseases of the spleen, where the white blood-corpuscles were increased. In this patient, the act of lying down, by increasing pressure on the diaphragm, induced the dyspnœa. Would add, that the woman was thirty-five years of age, and the mother of four or five children. Used tonic and sustaining treatment, on account of the debility.

The President, Dr. Logan, states that he has recently treated two patients that he is convinced were cases of typhoid fever. Most of the fever that he has seen was malarious; has seen some few cases, which he reported at a late meeting, of a type with which he was not familiar. Does not know that there is any tendency to a prevalence again of typhoid fever epidemic, but is confident—from the nervous symptoms, the duration of four week, etc.—that these were genuine cases of that disease. Has been informed that there were a number of hog-pens in the vicinity. Cannot say that this had any effect in producing the sickness, but mentions it as a matter that might throw some light on it. In the treatment, quinine was fully tested, without cutting short or ameliorating the disease. In the commencement, there was some periodicity, to a greater or less extent; this disappeared, and the fever continued its insidious march.

Dr. Wells asks, if delirium or mental derangement was present? Also, if there was any tympanites?

Dr. Logan.—There was some delirium in one. The friends reported some mental derangement in the other, though he did not see it. This was a less marked feature than was the nervous disturbance. As to abdominal symptoms, they were not so decided as usual, though, in the last, there was such a tendency. No decided diarrhœa in either case.

Dr. Withers.—Saw, twelve months ago, two well-marked cases of typhoid fever, one of them dying from hemorrhage from the bowels. Has recently seen some remittent, and two or three cases of a continued fever. In the latter, there was no diarrhœa, no tympanites, no characteristic appearance of the tongue; hence, does not think it was typhoid. It yielded to tonics, stimulants, etc.

Dr. Wells.—Has seen only one case that he decided to be typhoid. The symptoms were so marked as to justify an absolute diagnosis. True, there was absence of tympanites, and but little diarrhœa; but this, he thinks, may have been attributable to the use of turpentine. The disease progressed to the twenty-first day. It was followed by some interesting sequelœ, which he may report at some other time.

He would suggest that the intermittent symptoms, that some of the members have met with in these cases of continued fever, may have been owing to a really malarious influence. Such was his observation when typhoid fever prevailed extensively in a malarious region of Alabama. So much did it partake of the intermittent character as to sometimes entirely mislead the physician, till the fever had continued for a week or two despite treatment. Thinks that some localities of the city, from deficient draining and other causes, may generate sufficient malaria to complicate cases of fever.

Dr. J. G. Westmoreland is inclined to think that typhoid fever is again about to prevail among us. It is not necessary that malaria be present for the disease to present this intermittency. It may be a part of the disease itself. Has often met an intermission so perfect as not to suspect its true nature till medication had failed to produce the looked-for effect. Thinks we now have some typhoid in the city. An absence of the diarrhœa or delirium is not sufficient to exclude the diagnosis of typhoid; has seen many cases in which they were wanting.

Dr. Withers has never seen a case in which the abdominal trouble was lacking. A slight purgative will always bring on the diarrhœa. If there is no abdominal derangement, he always thinks it is some other disease than typhoid. Believes the bowels are always affected, and must produce some symptom.

Dr. J. G. Westmoreland.—Must still differ from Dr. Withers. Has seen not a few well-marked cases where the course was perfectly regular, in which this particular symptom did not appear—in which there was no symptom pointing to a bowel affection. The diarrhœa is usual, but is not an essential characteristic of the disease. Bronchitis is almost as frequently present as is diarrhœa.

Dr. Withers.—In determining the diagnosis, or in making a *post-mortem* examination, decides there is no typhoid if there is no evidence of the involvement of the intestinal glands.

Dr. J. G. Westmoreland.—You might decide wrong.

Dr. Withers.—Has seen cases, lasting three or four weeks, in which the patients did not take bed. Sometimes no diarrhœa is present till some medicine is given; the administration of a mild laxative will bring on the symptom. Possibly, if the case be let alone in that respect, there may be no diarrhœa.

Dr. J. G. Westmoreland.—Has seen such cases, where the cathartic initiated the diarrhœa. As to the intermission or remission spoken of, his experience proves, that while it may occur daily, it is not regular as to hours; but usually, the fever is coolest in the morning. Endeavors to avoid diarrhœa by refraining from the use of cathartics. Would suggest, that if Dr. Withers had been so careful as to cathartics, the diarrhœa might not have been so uniformly present under his observation.

Dr. W. H. Cumming.—Would ask Dr. Westmoreland if any of the cases in which diarrhœa or other evidence of bowel complication was absent, were severe?

Dr. Westmoreland.—Yes.

Dr. Cumming.—In the majority of cases that die, we may expect the bowel disease. In deciding as to the remission, the thermometer can be of service. The disease has been thus shown to have its regular variations of temperature in morning and evening. The tables of the variations are interesting to study. The progress toward convalescence may be determined, by the change in the relation of their variation, before other symptoms have announced it.

Typhoid fever has its mild cases; five times as many recover as die. 'Tis only the fatal ones wherein we can observe the pathological changes. In many well-marked cases, there is an absence of some of the symptoms. The fever poison seems to be of all grades. Some patients take heavier doses of it than others. Some are stricken down at once, as in severe cases of scarlet fever. Has observed cases in which there was neither diarrhœa or delirium; but there was great stupidity of the countenance; also, the decline of strength during and after the disease. Regards this latter as one of the best marks of typhoid fever.

Dr. J. G. Westmoreland.—Since most of the fatal cases die from the bowel affection, it is natural that we should find this lesion at the autopsy. This accounts for the belief in the bowel pathology. As the other cases do not usually die, we have no positive proof of the pathological changes.

Dr. Withers.—Holds that the bowel affection is as an essential characteristic as is the eruption in small-pox.

---

